# NASP Promotes Triple-negative Breast Cancer Progression and Metastasis by Stabilizing YAP in a USP15-Dependent Way

**DOI:** 10.7150/ijbs.99438

**Published:** 2025-06-20

**Authors:** Wenfang Zheng, Qifeng Luo, Xuehui Wang, Xiqian Zhou, Danrong Ye, Kaiyao Hua, Lin Fang

**Affiliations:** Department of Breast and Thyroid Surgery, Shanghai Tenth People's Hospital, School of Medicine, Tongji University, Shanghai, 200072, China.

**Keywords:** triple-negative breast cancer, NASP, USP15, YAP, metastasis

## Abstract

Triple-negative breast cancer (TNBC) was a subtype of breast cancer with high rate of metastasis and poor prognosis. Thus, it is urgent to explore the underlying mechanism of TNBC metastasis and seek for potential therapeutic targets to improve the prognosis of TNBC patients. Here we reported that nuclear autoantigenic sperm protein (NASP) was highly expressed in TNBC and related to poor prognosis of TNBC patients. NASP acted as an oncogene that promoted the progression and metastasis of TNBC. Mechanistically, high expression of NASP in TNBC was induced by SRSF1-mediated stabilization of NASP mRNA. NASP interacted with USP15 and facilitated its activity, which resulted in the deubiquitylation and stabilization of YAP by erasing K48-linked polyubiquitination. Moreover, in vivo studies validated the role of NASP in stimulating TNBC growth and metastasis. Altogether, NASP promoted TNBC progression and metastasis by stabilizing YAP in a USP15-dependent way. It might provide new insights and potential therapeutic targets for preventing TNBC metastasis and improving the prognosis of TNBC patients.

## Introduction

Breast cancer has become the most common malignancy worldwide, with the highest morbidity and mortality among women's malignancies 1. With the development of cancer therapies, the prognosis of early-stage breast cancer patients has been improved 2. However, some patients may suffer tumor metastasis due to therapeutic resistance, which is the main cause of breast cancer related death 3. Among the major molecular subtypes of breast cancer, triple-negative breast cancer (TNBC) lacks hormone receptors (HR) or human epidermal growth factor receptor-2 (HER-2), resulting in being short of therapeutic targets and tumor metastasis 4. Therefore, it is urgent to seek for novel therapeutic targets to improve the prognosis of TNBC patients.

Nuclear autoantigenic sperm protein (NASP) was first discovered in the nuclear area of primary spermatocytes and sperms 5. Previous studies indicated that NASP was a conserved H3-H4 histone chaperone and took part in the metabolism of histone 6. There were two major non-allelic isoforms of NASP in human, the testicular NASP (tNASP) and somatic NASP (sNASP) 7. tNASP was reported to be expressed in cancer, gametes, embryonic cells and transformed cells, while sNASP was found in all mitotic somatic cells 8, 9. NASP appeared to take part in the progression of several cancers. In hepatocellular carcinoma, NASP antagonized the chromatin accessibility and promoted tumorigenesis 10. Depletion of tNASP resulted in inhibition of prostate cancer 8. In breast cancer, sNASP was associated with 5-Fluorouracil sensitivity and the cellular sensitivity to 5-Fluorouracil was suppressed when sNASP was inhibited 11. However, the role of NASP in TNBC progression and metastasis is still elusive.

Ubiquitination is one of the post-translational modifications and it is crucial in multiple biological processes 12. The covalently links between target protein and the ubiquitin protein are mainly regulated by ubiquitin activating enzymes, ubiquitin conjugating enzyme and ubiquitin ligases 13, while the ubiquitination can be erased by deubiquitinating enzymes (DUBs) 14. Ubiquitin specific peptidases (USPs) belong to DUBs, and their roles in removing ubiquitin from proteins pretend proteins from degradation 15. An increasing number of studies have demonstrated that USPs are vital in tumorigenesis and malignant phenotypes of tumor 16. For instance, USP22, USP36, and USP37 regulate cancer progression by stabilizing oncogenic c-Myc 17-19. USP4 promotes metastasis of hepatocellular carcinoma via stabilizing TGFβ receptor type 1 20. USP10 deubiquitinates and protects IGF2BP1 to assisting breast cancer metastasis 21.

Here we discovered that NASP was overexpressed in TNBC, and high expression of NASP was correlated to metastasis of TNBC. Serine and arginine rich splicing factor 1 (SRSF1)-mediated NASP formed a complex with USP15 and facilitated USP15 to deubiquitinate and stabilize yes-associated protein (YAP), thereby promoting TNBC metastasis.

## Materials and Methods

### Bioinformatic analysis

GEPIA2 (http://gepia2.cancer-pku.cn/#index) was used to analyze the expression of NASP in breast cancer. Kaplan-Meier plotter (https://kmplot.com/analysis/) was used to evaluate the relationship between NASP expression and distant metastasis-free survival (DMFS). The human protein atlas (https://www.proteinatlas.org) was used to assess the protein levels of NASP in breast cancer. BioGRID (https://thebiogrid.org/) was used to explore the potential protein-protein interactions.

### Patients and clinical samples

A total of 80 TNBC patients from Shanghai Tenth People's Hospital were involved in this study. All patients were newly diagnosed with TNBC and they had not received any anti-tumor therapies before surgery. Tumor tissues and paired adjacent tissues were obtained immediately after the surgery. For patients with distant metastasis, the metastatic tissues were collected by biopsy. The clinicopathological information of involved patients were also collected. This study was approved by the Ethics Committee of Shanghai Tenth People's Hospital.

### Cell culture, cell transfection and lentivirus infection

TNBC cell lines (MDA-MB-231, BT549 and MDA-MB-468) and non-tumorigenic breast epithelial cell line (MCF-10A) from the Chinese Academy of Sciences were used in this study. The cell culture conditions were the same as previous study 22. SiRNAs (IBSbio, Shanghai, China) were used for cell transfection and their sequences were showed in Table S1. Myc-tagged USP15 and its mutants, HA-tagged ubiquitin and its mutants (K48R and K63R), Flag-tagged SRSF1 and its truncations, and GFP-fused YAP and its truncations were cloned to pcDNA3.1. Cell transfection was conducted using Lipofectamine^®^ 3000 reagent (Invitrogen, USA). To establish the cell lines that stably overexpress NASP, the lentiviral plasmids was constructed using pCDH-MSCV-MCS-EF1-GFP-puro vector and then packaged by GMeasy Lentiviral Packaging Kit (Genomeditech, Shanghai, China). After infection, the cells were chosen by 1μg/mL puromycin (Beyotime, Shanghai, China). To establish the cell lines that stably knockdown NASP with shRNA, the lentivirus carrying sh-NASP was constructed by Miaoling biology (Wuhan, China) and the procedures of infection and selection were performed as described above. The sequence of sh-NASP was 5′-GGAAATCACTTCTGGAGTTttcaagagaAACTCCAGAAGTGATTTCC-3′.

### MTT assay, colony formation assay, wound-healing assay and Transwell assay

MTT assay and colony formation assay were conducted to evaluate the proliferation of TNBC cells. Wound healing assay and Transwell assay were performed to detect the migration of TNBC cells. The procedures of these experiments were as previously described 22.

### RNA extraction, reverse transcription (RT), polymerase chain reaction (PCR) and real time quantitative PCR (RT-qPCR)

The procedures of RNA extraction, RT, PCR and RT-qPCR were described in previous study 22. The primers used for these procedures were listed in Table S2.

### Protein extraction and western blotting

These procedures were described in previous study 22. The antibodies and their dilutions were as follows: anti-NASP (1:1000, 11323-1-AP, proteintech, USA), anti-USP15 (1:1000, 14354-1-AP, proteintech, USA), anti-YAP1 (1:1000, A21216, ABclonal, China), anti-CTGF (1:1000, A11456, ABclonal, China), anti-ubiquitin (1:1000, sc-8017, Santa Cruz Biotechnology, USA), anti-GFP (1:1000, sc-9996, Santa Cruz Biotechnology, USA), anti-β-Actin (1:1000, sc-47778, Santa Cruz Biotechnology, USA), anti-HA (1:1000, sc-7392, Santa Cruz Biotechnology, USA), anti-myc-tag (1:1000, 16286-1-AP, proteintech, USA), Dylight 800-goat anti-rabbit IgG (1:2000, A23920, Abbkine, USA), Dylight 800-goat anti-mouse IgG (1:2000, A23910, Abbkine, USA), and HRP Goat Anti-Mouse IgG (H+L) (1:1000, AS003, ABclonal, China).

### Actinomycin D, MG-132 and cycloheximide (CHX) treatment

For actinomycin D assay, the cells were treated with 1μg/mL actinomycin D (YEASEN, Shanghai, China) for 0h, 4h, 8h, 12h. For MG-132 treatment, the cells were incubated with 10μM MG-132 for 4h. For CHX treatment, the cells were treated with 20μg/mL CHX for 0h, 4h, 8h, 16h before protein extraction.

### RNA immunoprecipitation (RIP)

RNA Immunoprecipitation Kit was used for RIP assay following the manufacturer's instructions. Anti-Flag (AE005, Abclonal, China) was used for RIP assay. The products of RIP assay were analyzed by RT-qPCR and agarose gel electrophoresis.

### Coimmunoprecipitation (co-IP)

The procedure of co-IP referred to previous study 22. Antibodies used in this experiment were as follows: anti-YAP1 (A21216, ABclonal, China), anti-USP15 (14354-1-AP, proteintech, USA), anti-GFP (sc-9996, Santa Cruz Biotechnology, USA), and anti-myc-tag (16286-1-AP, proteintech, USA).

### Immunofluorescence (IF)

The procedure of IF assay was described in previous study 22. Anti-YAP1 (sc-376830, Santa Cruz Biotechnology, USA), anti-USP15 (14354-1-AP, proteintech, USA), Alexa Fluor 488-goat anti-mouse IgG(H+L) (YEASEN, Shanghai, China), and Cy3-goat anti-mouse IgG(H+L) (YEASEN, Shanghai, China). were used for IF assay. Nucleus were stained with DAPI.

### Immunohistochemistry (IHC) and hematoxylin and eosin (H&E) staining

For IHC, after fixation, dehydration, embedment and slicing, the sections were stained with anti-NASP (11323-1-AP, proteintech, USA). For H&E staining, the sections were stained with H&E.

### Animal experiments

For xenografts experiment, BALB/c nude mice (4-week-old, female, SLAC, China) were randomly divided into four groups (n=5 for each group). 2×10^6^ BT549 cells stably expressing sh-NC, sh-NASP, LV-vector and LV-NASP were injected subcutaneously to each group respectively. All mice injected with sh-NC or sh-NASP cells were sacrificed after 10 weeks, while mice injected with LV-vector and LV-NASP cells were sacrificed after 6 weeks. The tumors were then collected. Tail vein injection was used to establish the lung metastasis model and 6-week-old female BALB/c nude mice were employed. 2×10^6^ BT549 cells were injected into tail veins of mice. All mice injected with sh-NC or sh-NASP cells were sacrificed after 8 weeks, while mice injected with LV-vector and LV-NASP cells were sacrificed after 4 weeks. To establish the liver metastasis model, intrasplenic injection (2×10^6^ BT549 cells/mouse) was performed on 6-week-old female BALB/c nude mice after anesthetizing mice with avertin. All mice injected with sh-NC or sh-NASP cells were sacrificed after 8 weeks, while mice injected with LV-vector and LV-NASP cells were sacrificed after 3 weeks. Tissues of mice were collected.

### Statistical analysis

GraphPad Prism 8 (GraphPad Software, USA) and SPSS Statistics 20 (IBM, USA) were used for data analysis. All data were presented as mean±standard deviation (SD). Data of paired specimens was analyzed by Wilcoxon matched-pairs signed rank test. Unpaired Student's t-test was used to analyze data of unpaired samples. Log rank test was employed to evaluate the progression-free survival (PFS). The relationships between NASP and clinicopathological information was assessed by χ^2^ test. Two-way ANOVA was used to analyze the data of MTT assay and actinomycin D treatment. Pearson's correlation coefficient was conducted to explore the correlation between NASP and SRSF1. All experiments were independently performed at least three times. *P*<0.05 was considered statistically significant.

## Results

### NASP was upregulated in TNBC and related to TNBC metastasis

**Figure 1:**
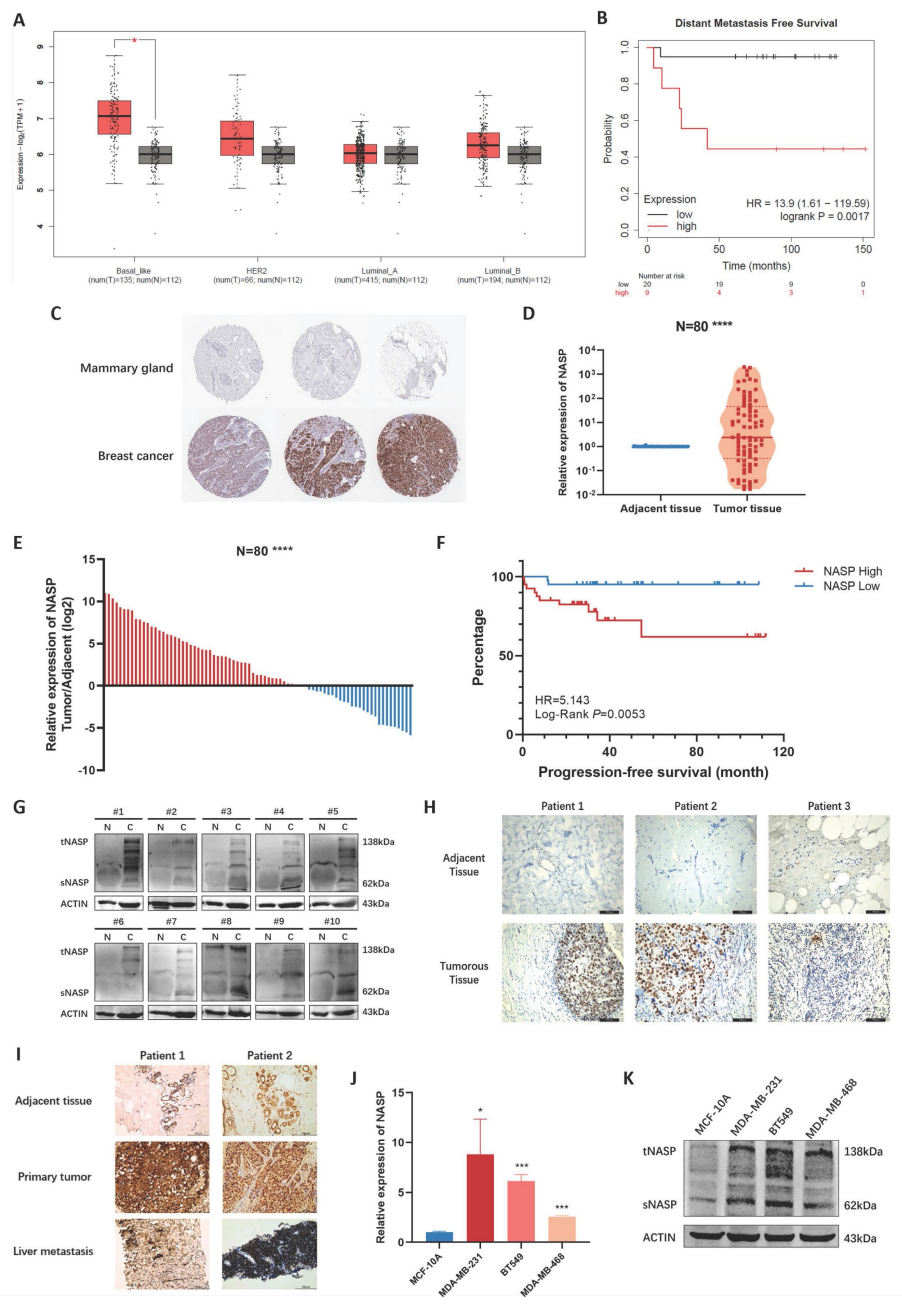
NASP was highly expressed in TNBC. **A** The expression of NASP in different subtypes of breast cancer in GEPIA2 database. **B** The analysis of NASP expression and DMFS by K-M plotter. **C** The IHC analysis of NASP expression in breast cancer in the Human Protein Atlas. **D** The expression of NASP in TNBC tissues and paired adjacent tissues detected by RT-qPCR (N=80). **E** The analysis of NASP expression in TNBC tissues compared to adjacent tissues (N=80). **F** The K-M plot to analyze the relationship between NASP expression and PFS in TNBC patients. **G** The expression of NASP in TNBC tissues and paired adjacent tissues detected by western blotting. **H** IHC analysis of NASP expression in TNBC tissues and paired adjacent tissues. **I** IHC analysis of NASP expression in TNBC tissues, paired adjacent tissues and liver metastatic lesions. **J** NASP expression in TNBC cells detected by RT-qPCR. **K** The expression of NASP protein in TNBC cells detected by western blotting. *P<0.05, **P<0.01, ***P<0.001, ****P<0.0001, ns: no significance.

The expression and clinical significance were firstly analyzed in databases. By exploring GEPIA2 database, we noticed that NASP was highly expressed in TNBC, while there were no significant differences in other subtypes of breast cancer (Figure 1A). The result of K-M plotter analysis demonstrated that patients with high NASP expression were more likely to have distant metastasis (Figure 1B). In addition, the IHC analysis in the Human Protein Atlas showed that the expression of NASP protein was higher in breast cancer tissue than in mammary gland (Figure 1C). We next detected the expression of NASP in collected specimens of TNBC patients (N=80). The results of RT-qPCR suggested that NASP expression was significantly higher in tumor tissues than in paired adjacent tissues (Figure 1D, 1E). According to the median of NASP expression, the patients were divided into high expression group and low expression group. The relationships between NASP expression and the clinical characteristics of TNBC patients were then analyzed. The results presented in Table 1 demonstrated that NASP expression was positively correlated to TNBC metastasis. Furthermore, the PFS of high NASP expression group was significantly shorter than low NASP expression group, indicating that high expression of NASP was related to poor prognosis in TNBC patients (Figure 1F). The protein level of NASP in TNBC was also detected by western blotting and IHC. The results showed that compared to paired adjacent tissues, NASP protein was highly expressed in TNBC tumor tissues (Figure 1G, 1H). The NASP expression was then assessed by IHC in the adjacent tissues, primary tumor tissues and liver metastases of two patients with TNBC metastasis. The intensity of NASP was higher in primary tumor tissues and metastatic lesions than in adjacent tissue (Figure 1I). Besides, we analyzed the expression of NASP in TNBC cell lines and discovered that NASP was highly expressed in TNBC cell lines than in MCF-10A (Figure 1J, 1K). Besides, the expression of NASP did not significantly elevated or depleted in SKBR3 (represents Her2+ breast cancer cells) or MCF-7 (represents luminal breast cancer cells) cell lines (Figure S1), which was consistent with the result from GEPIA2 database showed in Figure 1A. Collectively, these results suggested that NASP was highly expressed in TNBC and related to poor prognosis.

### NASP played an oncogenic role in TNBC

**Figure 2:**
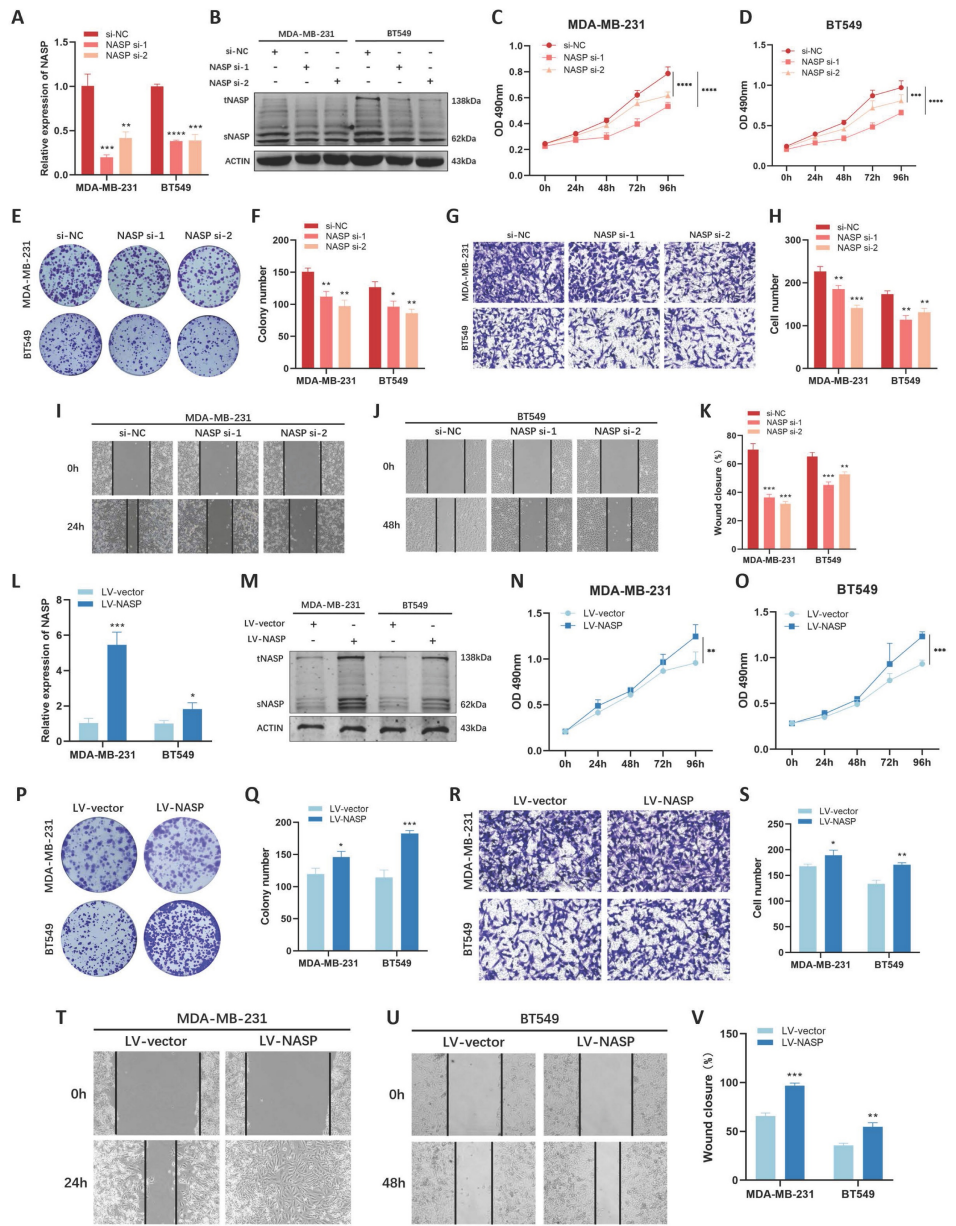
NASP played an oncogenic role in TNBC cells. **A** The efficiency of NASP siRNAs detected by RT-qPCR. **B** The expression of NASP protein in NASP siRNA-transfected TNBC cells detected by western blotting. **C** The proliferation of MDA-MB-231 cells transfected with NASP siRNAs detected by MTT assay. **D** The proliferation of BT549 cells transfected with NASP siRNAs detected by MTT assay. **E** Colony formation assay showing the colonies of NASP siRNA-transfected TNBC cells. **F** The number of colonies. **G** Transwell assay comparing the migration of NASP siRNA-transfected TNBC cells. **H** The number of migrated cells in Transwell assay. **I** Wound-healing assay showing the migration of NASP siRNA-transfected MDA-MB-231 cells. **J** Wound-healing assay showing the migration of NASP siRNA-transfected BT549 cells. **K** The statistics of wound-healing assay. **L** The efficiency of LV-NASP in TNBC cells detected by RT-qPCR. **M** The expression of NASP protein in TNBC cells in LV-vector and LV-NASP groups detected by western blotting. **N** The proliferation of MDA-MB-231 cells in LV-vector and LV-NASP groups analyzed by MTT assay. **O** The proliferation of BT549 cells in LV-vector and LV-NASP groups analyzed by MTT assay. **P** The colony formation assay showing the colonies in LV-vector and LV-NASP groups of TNBC cells. **Q** The number of colonies. **R** Transwell assay showing the migration of TNBC cells in LV-vector and LV-NASP groups. **S** The number of migrated TNBC cells in Transwell assay. **T** Wound-healing assay showing the effect of LV-NASP on the migration of MDA-MB-231 cells. **U** Wound-healing assay showing the effect of LV-NASP on the migration of BT549 cells. **V** The statistics of wound-healing assay. *P<0.05, **P<0.01, ***P<0.001, ****P<0.0001.

To explore the biological functions of NASP in TNBC, we conducted functional analysis in vitro. We first used siRNAs to interfering the expression of NASP in TNBC cells and detected the efficiency of siRNAs by RT-qPCR (Figure 2A) and western blotting (Figure 2B). After transfecting siRNAs, MTT assay was performed and the results showed that knocking-down of NASP suppressed the proliferation of MDA-MB-231 cells and BT549 cells (Figure 2C, 2D). The results of colony formation assay showed that the colony formation ability decreased when NASP was suppressed (Figure 2E, 2F). Transwell assay and wound-healing assay were conducted to check the migration of TNBC cells, and the results demonstrated that the migration of MDA-MB-231 cells and BT549 cells was significantly inhibited after transfecting siRNAs of NASP (Figure 2G-2K).

We also analyzed the effect of overexpressing NASP on cell proliferation and migration in TNBC. By using lentiviral infection, the TNBC cell lines which stably overexpressing NASP were established (Figure 2L, 2M). The results of MTT assay, colony formation assay, Transwell assay and wound-healing assay suggested that overexpressing NASP promoted the proliferation and migration of MDA-MB-231 and BT549 cells (Figure 2N-2V). Taken together, NASP acted as an oncogene in TNBC.

### SRSF1 promoted NASP expression by stabilizing NASP mRNA

**Figure 3:**
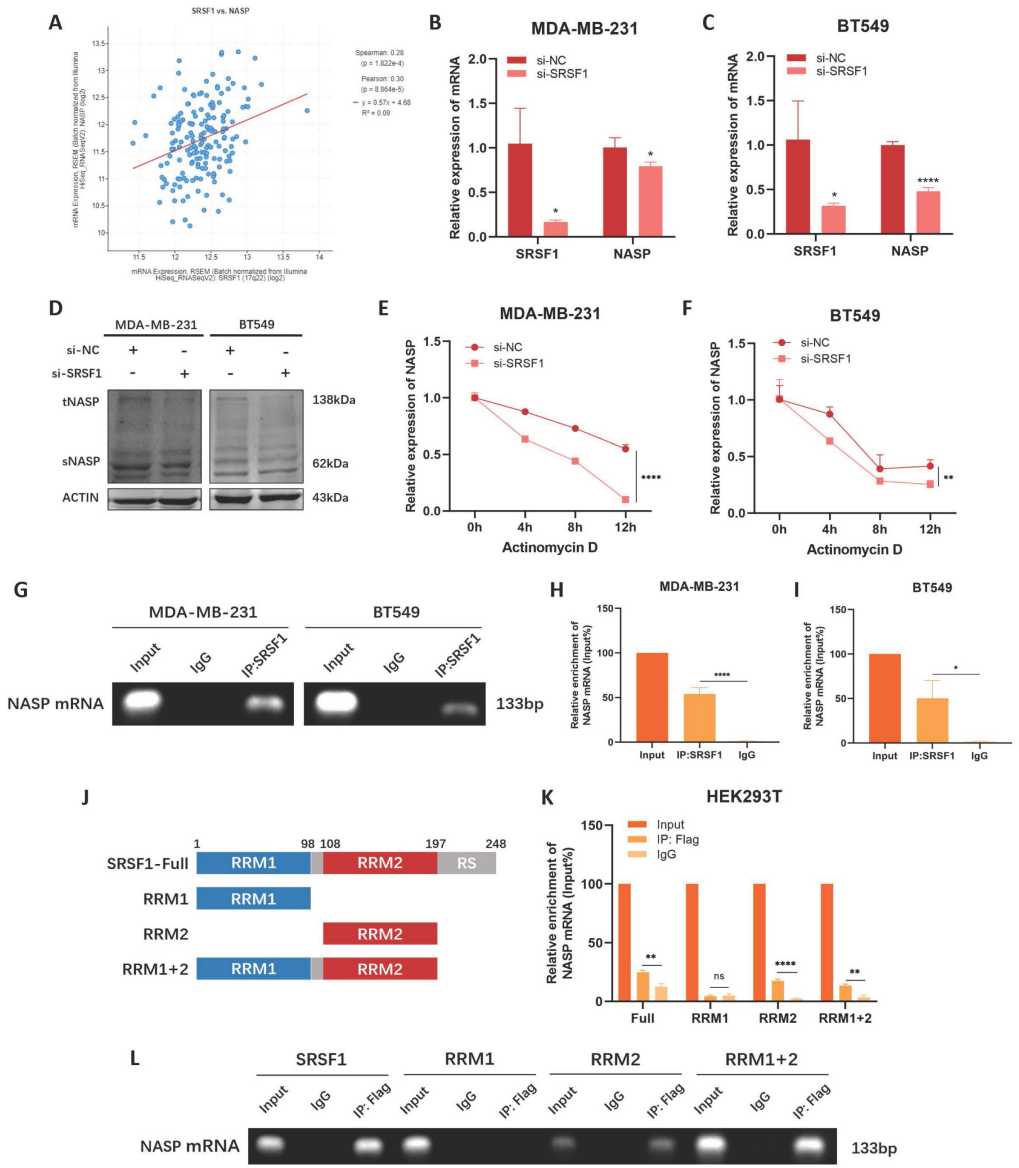
SRSF1 promoted NASP expression in TNBC by facilitating the stability of NASP mRNA. **A** The correlation between SRSF1 and NASP expression in breast cancer. **B** The expression of SRSF1 and NASP mRNA in MDA-MB-231 cells after transfecting si-SRSF1 detected by RT-qPCR. **C** The expression of SRSF1 and NASP mRNA in BT549 cells after transfecting si-SRSF1. **D** The expression of NASP protein detected by western blotting after transfecting si-SRSF1 in TNBC cells. **E** RT-qPCR determining the expression of NASP mRNA after being treated with actinomycin D for 0h, 4h, 8h and 12h in si-NC and si-SRSF1 groups of MDA-MB-231 cells. **F** RT-qPCR determining the expression of NASP mRNA after being treated with actinomycin D for 0h, 4h, 8h and 12h in si-NC and si-SRSF1 groups of BT549 cells. **G** Agarose gel electrophoresis analysis of the products of RIP assay. **H** RT-qPCR detecting the enrichment of NASP mRNA in the products of RIP assay from MDA-MB-231 cells. **I** RT-qPCR detecting the enrichment of NASP mRNA in the products of RIP assay from BT549 cells. **J** Schematic illustration showing the structure of SRSF1. **K** RT-qPCR detecting the enrichment of NASP mRNA in the products of RIP assay after transfecting SRSF1 mutants into HEK293T cells. **L** Agarose gel electrophoresis analysis of the products of RIP assay after transfecting SRSF1 mutants into HEK293T cells. *P<0.05, **P<0.01, ***P<0.001, ****P<0.0001.

Considering there was abnormal increase of NASP expression in TNBC, we explored the potential reason. SRSF1 was an RNA binding protein and it played an important role in RNA metabolism 23. It participates in regulating alternative splicing 24, mRNA stabilization 25, and mRNA translation 26. We noticed that the expression of NASP was positively correlated to SRSF1 expression in TNBC (Figure 3A). Thus, we investigated the role of SRSF1 in NASP expression. When SRSF1 expression was inhibited by siRNA, the mRNA and protein levels of NASP were both decreased in TNBC cells (Figure 3B-3D). Therefore, we hypothesized that SRSF1 could regulate the mRNA level of NASP by stabilizing NASP mRNA. To validate this hypothesis, we treated TNBC cells with actinomycin D to detect the effect of SRSF1 on the half-life period of NASP mRNA. We noticed that the half-life period of NASP mRNA was shorter when SRSF1 was suppressed, indicating that SRSF1 promoted the stability of NASP mRNA (Figure 3E, 3F). We next performed RIP assay to explore whether there was an interaction between NASP mRNA and SRSF1. The results of RIP assay showed that NASP mRNA could be enriched by anti-SRSF1, demonstrated that SRSF1-NASP mRNA interaction existed in TNBC cells (Figure 3G-3I). Previous studies reported that SRSF1 contained two RNA-recognition motifs (RRM1 and RRM2) which could interact with RNA 27. We constructed the Flag-tagged truncation of SRSF1 and discovered that NASP mRNA could not be enriched when the RRM2 motif was deleted, suggesting that the formation of NASP mRNA-SRSF1 complex relied on the RRM2 motif (Figure 3J-3L). Overall, SRSF1 facilitated NASP expression by interacting with NASP mRNA and promoting its stabilization.

### NASP promoted the deubiquitylation of YAP via interacting with USP15

**Figure 4:**
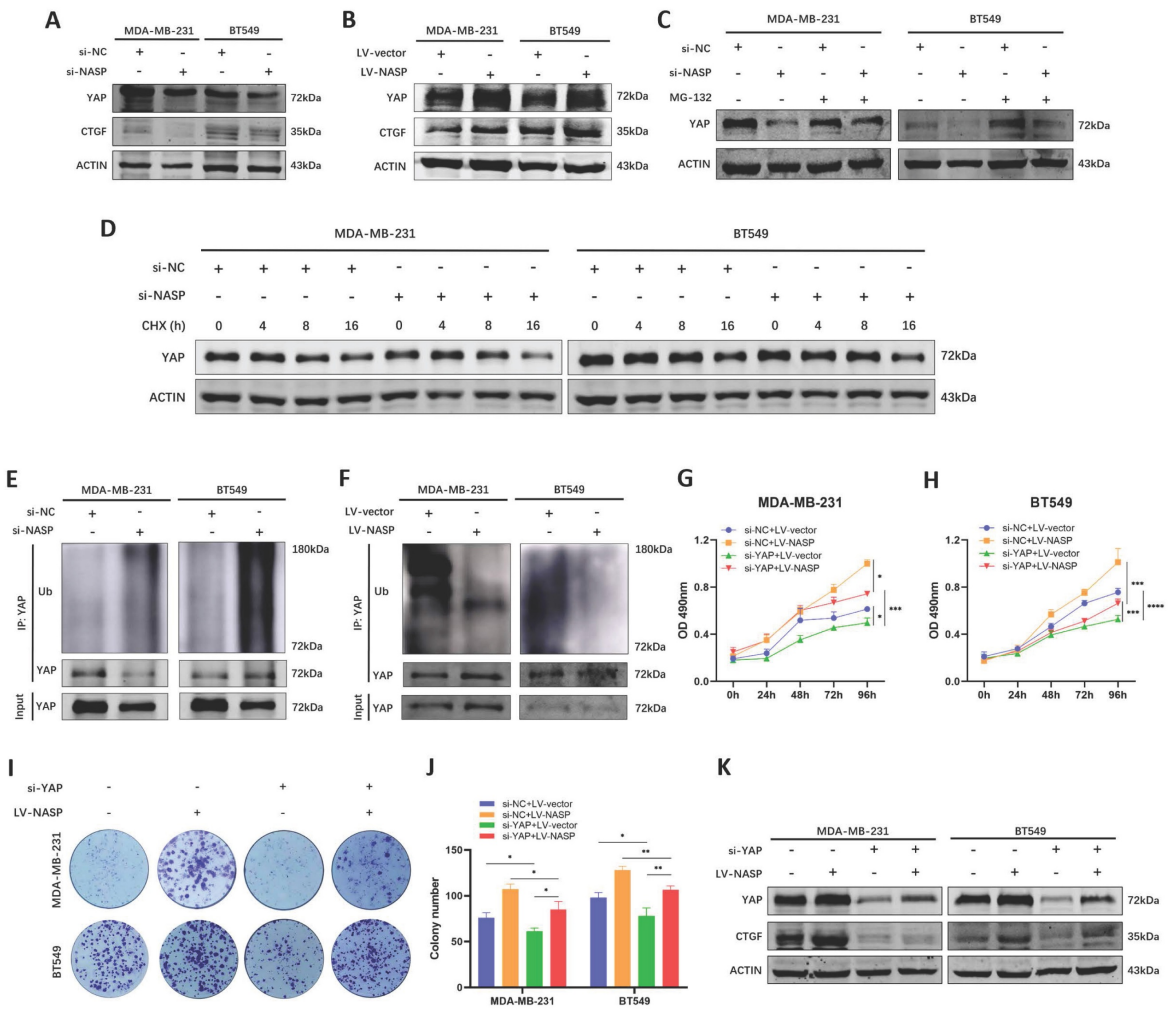
NASP promoted the stability of YAP by affecting its ubiquitination. **A** The expression of YAP and CTGF in TNBC cells after transfecting si-NASP. **B** The expression of YAP and CTGF in TNBC cells after overexpressing NASP. **C** The effect of MG-132 treatment on the expression of YAP in si-NC and si-NASP groups. **D** The half-life period of YAP in si-NC and si-NASP groups detected after treating with CHX for 0, 4, 8, 16h. **E** The effect of si-NASP on the ubiquitination of YAP in TNBC cells. **F** The effect of overexpressing NASP on the ubiquitination of YAP in TNBC cells. **G** MTT assay showing the influence of si-NASP on the proliferation of YAP-inhibited MDA-MB-231 cells. **H** MTT assay showing the influence of si-NASP on the proliferation of YAP-inhibited BT549 cells. **I** Colony formation assay showing the influence of si-NASP on the proliferation of YAP-inhibited TNBC cells. **J** The number of colonies. **K** The influence of si-NASP on the expression of YAP and CTGF in YAP-inhibited TNBC cells. *P<0.05, **P<0.01, ***P<0.001, ****P<0.0001.

YAP is an important transcriptional coactivator which activates the transcription of its downstream target genes after nuclear translocation 28. However, the phosphorylated YAP will undergo ubiquitination and degradation via ubiquitin-proteasome pathway 29. A plenty of studies had demonstrated that YAP was a key regulator in the process of tumor initiation 30, proliferation 31 and metastasis 32, and overexpression of YAP was related to poor prognosis of cancer patients 33. In our previous studies, we have reported the oncogenic role of YAP in breast cancer 34, 35. Therefore, we analyzed whether NASP was a regulator of YAP in TNBC. After inhibiting NASP, there was no significant change of the mRNA level of YAP in TNBC cells (Figure S2A). Accordingly, the overexpression of NASP did not change the expression of YAP mRNA either (Figure S2B). However, the protein levels of YAP and CTGF was downregulated after transfecting si-NASP, while overexpressing NASP elevated them (Figure 4A, 4B). These results raised the possibility that NASP affected YAP expression on the protein level. To further investigating the underlying mechanism of NASP regulating YAP, we treated TNBC cells with MG-132 and CHX, respectively. MG-132 is a proteasome inhibitor that prevents the proteasome substrates from degradation 36. CHX can inhibit protein synthesis in eukaryotic cells and it is used to determine the half-life period of proteins 37. The results showed that MG-132 partially eliminated the degradation of YAP induced by si-NASP in TNBC cells (Figure 4C). In addition, CHX treatment showed that suppressing NASP decreased the half-life period of YAP (Figure 4D). Further analysis demonstrated that the ubiquitination of YAP protein was increased by si-NASP (Figure 4E) and decreased by NASP overexpression (Figure 4F), raising the possibility that NASP might promote the stability of YAP via regulating its deubiquitylation. Rescue experiments also demonstrated that the oncogenic role of NASP in TNBC was dependent on YAP (Figure 4G-4K).

**Figure 5:**
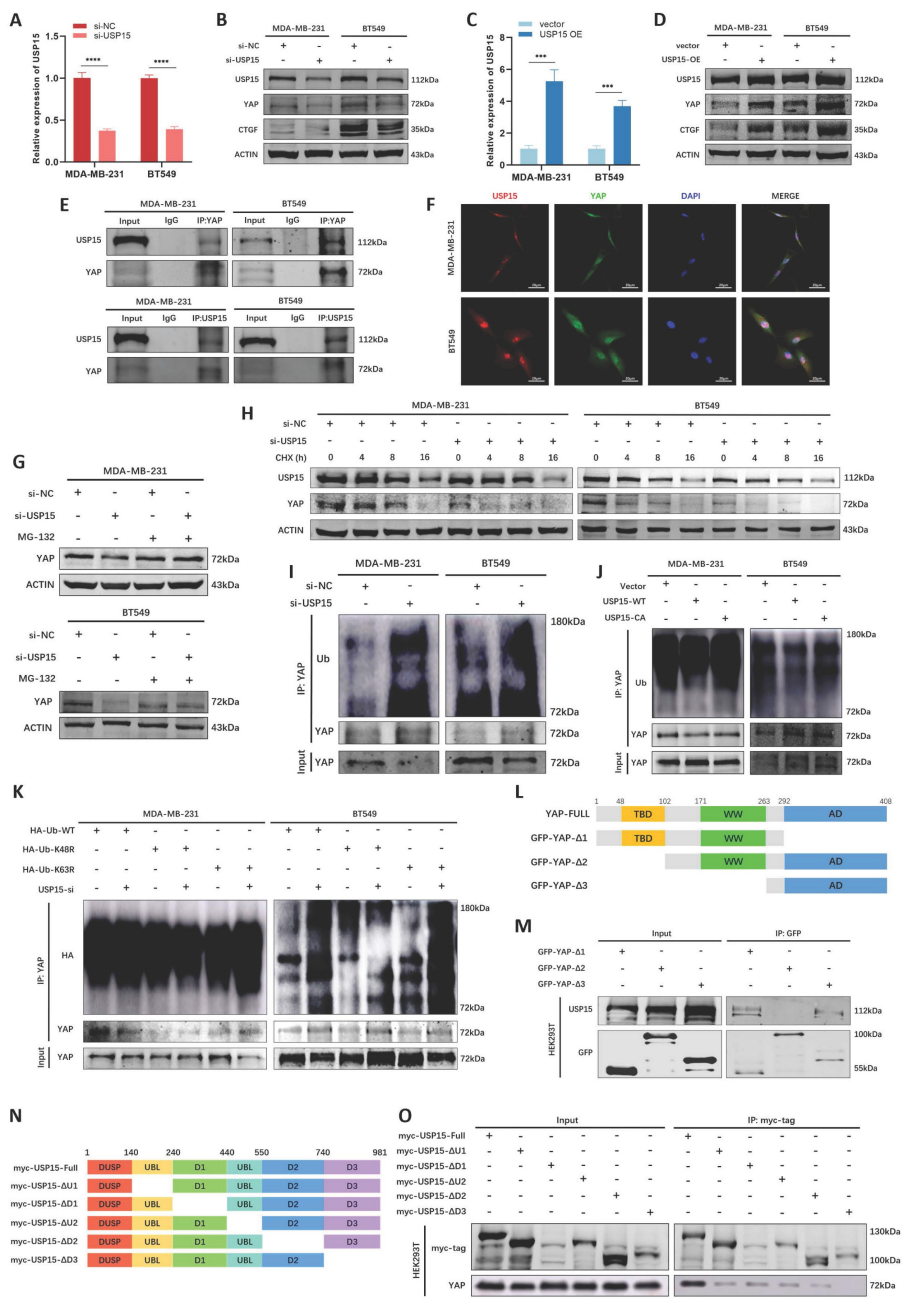
USP15 stabilized YAP in TNBC cells by deubiquitylating YAP. **A** The efficiency of si-USP15 in TNBC cells detected by RT-qPCR. **B** The expression of USP15, YAP and CTGF in TNBC cells after interfering USP15. **C** The efficiency of overexpressing USP15 in TNBC cells detected by RT-qPCR. **D** The expression of USP15, YAP and CTGF in TNBC cells after overexpressing USP15. **E** Co-IP assay analyzing the interaction between USP15 and YAP in TNBC cells. **F** IF assay detecting the co-localization of USP15 and YAP in TNBC cells. **G** The effect of MG-132 treatment on the expression of YAP in si-NC and si-USP15 groups. **H** The half-life period of YAP in si-NC and si-USP15 groups detected after treating with CHX for 0, 4, 8, 16h. **I** The effect of si-USP15 on the ubiquitination of YAP in TNBC cells. **J** The effect of overexpressing USP15-WT or USP15-CA on the ubiquitination of YAP in TNBC cells. **K** The type of polyubiquitylation affected by USP15 in TNBC. **L** Schematic illustration showing the structure of YAP. **M** The domain of YAP interacting with USP15 detected by co-IP assay. **N** Schematic illustration showing the structure of USP15. **O** The domain of USP15 interacting with YAP detected by co-IP assay. *P<0.05, **P<0.01, ***P<0.001, ****P<0.0001.

We then explored the potential interactors of NASP protein and YAP protein in BioGRID database, especially ubiquitination or deubiquitylation-related enzymes. We noticed that USP15 might be the interactor of both NASP and YAP (Figure S3A), thus we inferred that USP15 might be involved in NASP-mediated deubiquitylation of YAP. USP15 had been reported to deubiquitinate a variety of substrates 38-40. In MDA-MB-231 and BT549 cells, interfering or overexpressing USP15 did not affect the mRNA level of YAP (Figure S3B, S3C), but USP15 elevated the protein levels of YAP and downstream CTGF (Figure 5A-5D). Co-IP assay demonstrated that there was the interaction between YAP and USP15 (Figure 5E). Further IF assay showed the co-localization of YAP and USP15 in MDA-MB-231 and BT549 cells (Figure 5F), suggesting that USP15-YAP interaction existed in TNBC cells. In addition, MG-132 treatment partially eliminated the effect of inhibiting USP15 on YAP degradation (Figure 5G). CHX treatment showed that suppressing USP15 in TNBC cells fostered the degradation of YAP (Figure 5H). The ubiquitination of YAP protein was then detected. As showed in Figure 5I and Figure 5J, si-USP15 upregulated the ubiquitination of YAP, while wild-type USP15 (WT), but not enzymatic inactive mutant USP15^C269A^ 41, catalyzed the deubiquitylation of YAP. Previous studies reported that YAP was the substrate of lysine 48 (K48)- and K63-linked polyubiquitination 42, 43. Thus, we explored the type of polyubiquitin chains and discovered that suppressing USP15 could not elevate the polyubiquitination of YAP when K48 was mutated, demonstrating that USP15 catalyzed the K48-linked deubiquitylation of YAP (Figure 5K). Besides, the binding domain of USP15 and YAP were also analyzed. The results presented that the TBD domain of YAP interacted with the D3 domain of USP15 (Figure 5L-5O). Taken together, USP15 stabilized YAP in TNBC cells by removing K48-linked polyubiquitin chains from YAP.

**Figure 6:**
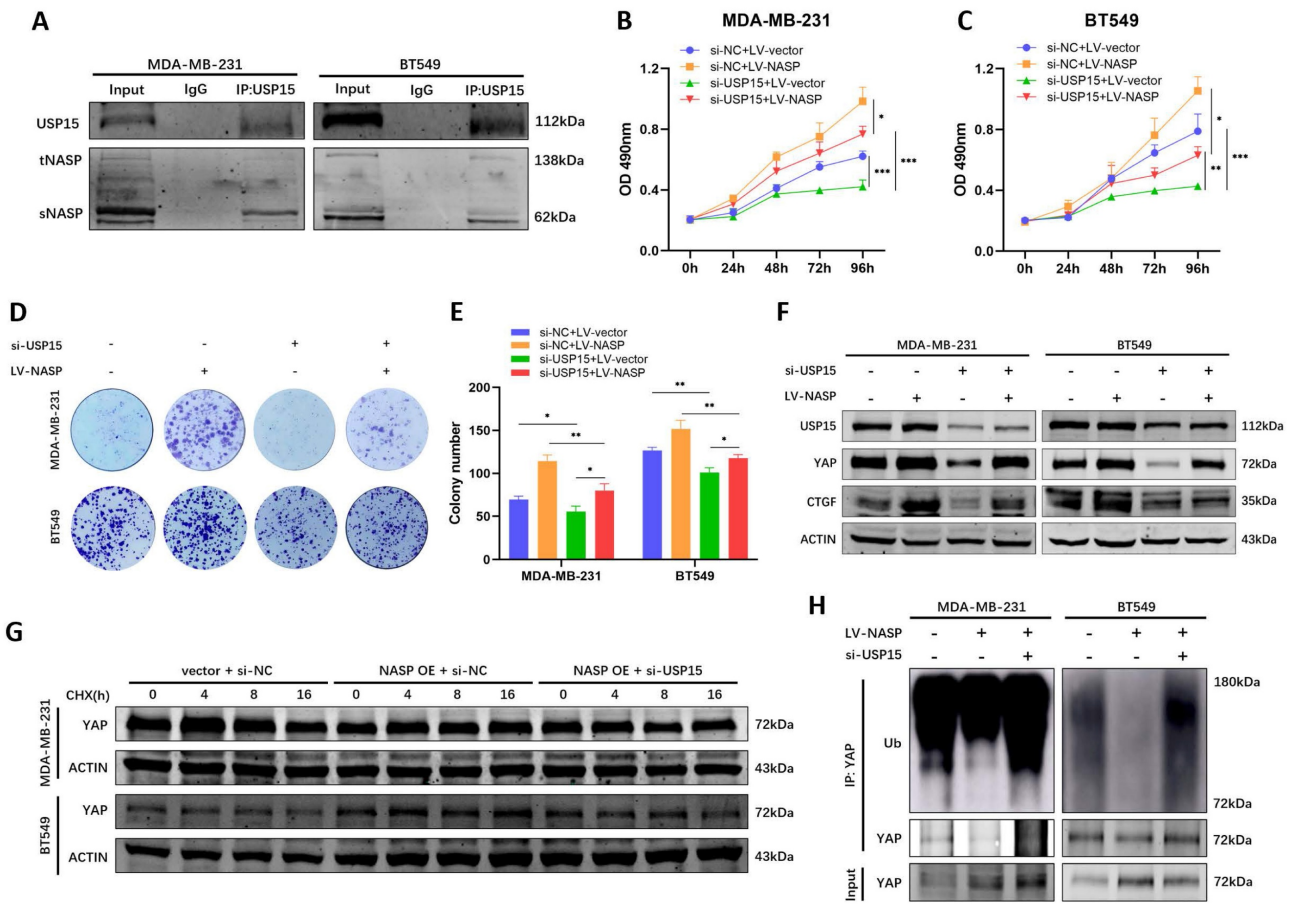
NASP regulated YAP in a USP15-dependent way. **A** Co-IP assay detecting the interaction between USP15 and NASP in TNBC cells. **B** MTT assay showing the influence of LV-NASP on the proliferation of USP15-inhibited MDA-MB-231 cells. **C** MTT assay showing the influence of LV-NASP on the proliferation of USP15-inhibited BT549 cells. **D** Colony formation assay showing the influence of LV-NASP on the proliferation of USP15-inhibited TNBC cells. **E** The number of colonies. **F** The influence of LV-NASP on the expression of USP15, YAP and CTGF in YAP-inhibited TNBC cells. **G** The effect of knocking down USP15 on YAP protein degradation in NASP-overexpressed TNBC cells after CHX treatment. **H** The effect of knocking down USP15 on YAP ubiquitination in NASP-overexpressed TNBC cells. *P<0.05, **P<0.01, ***P<0.001, ****P<0.0001.

To validate that NASP regulate the deubiquitylation of YAP in the USP15-dependent way, whether there was NASP-USP15 interaction in TNBC was first detected. Co-IP assay indicated that NASP could form complex with USP15 (Figure 6A). The following functional analyses demonstrated that suppressing USP15 in TNBC cells partially rescued the oncogenic role of NASP (Figure 6B-6E). Western blotting also indicated that inhibition of USP15 partially eliminated the effect of NASP on promoting expression of YAP and CTGF (Figure 6F). In addition, compared to overexpressing NASP alone, knocking down USP15 in NASP- overexpressed cells shortened the half-life period of YAP (Figure 6G). As for the ubiquitination of YAP, si-USP15 could block the deubiquitylation of YAP induced by NASP expression (Figure 6H). In short, NASP promoted the deubiquitylation of YAP in a USP15-dependent way.

### NASP promoted tumor growth and metastasis of TNBC *in vivo*

**Figure 7:**
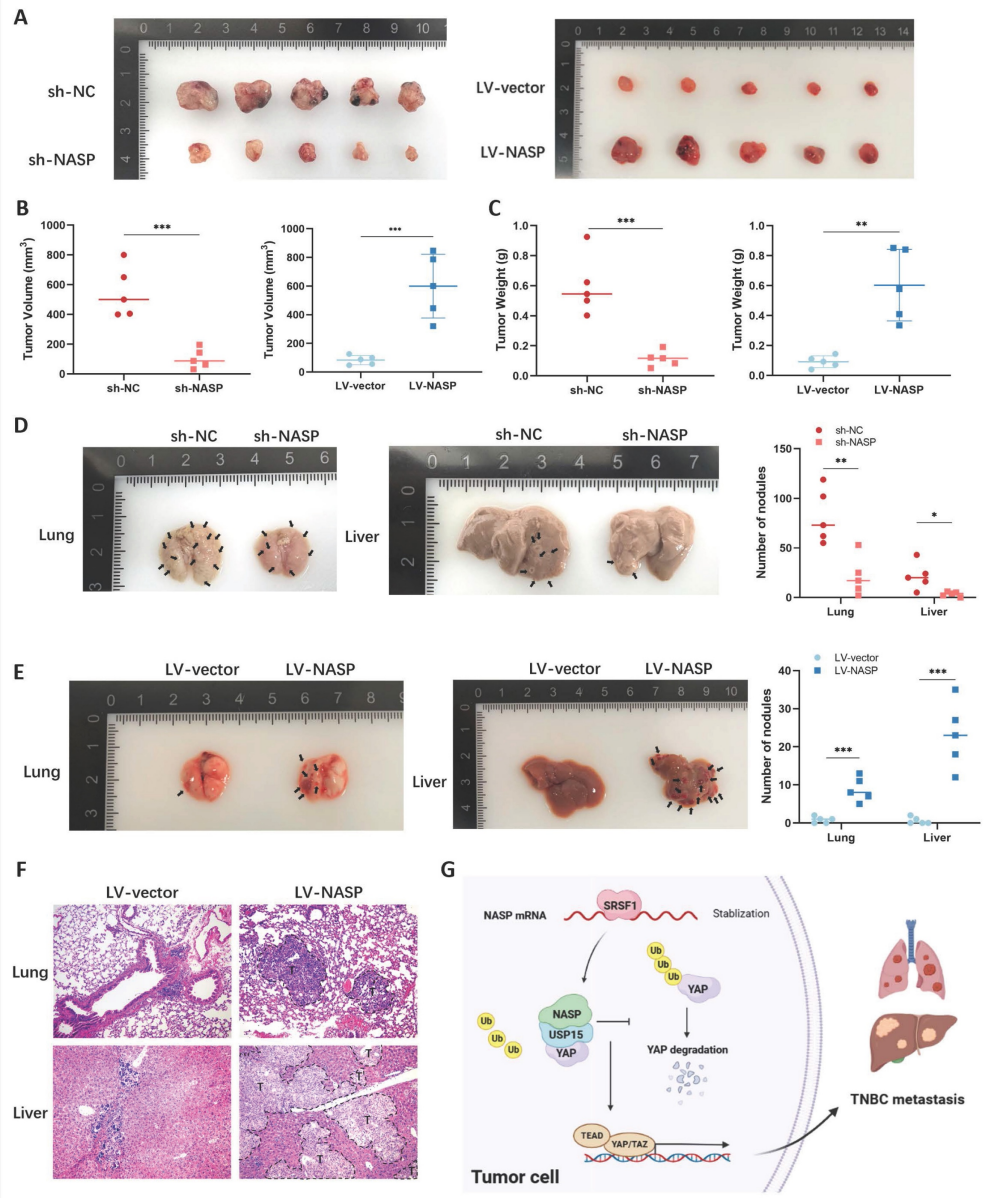
NASP promoted TNBC growth and metastasis in vivo. **A** Photograph of tumors in female BALB/c nude mice injected with sh-NC or sh-NASP BT549 cells (N=5 for each group) in left panel and photograph of tumors in female BALB/c nude mice injected with LV-vector or LV-NASP BT549 cells (N=5 for each group) in left panel. **B** The calculated volume of tumors in each group. **C** The weight of tumors in each group. **D** Photograph of lungs (left panel) and livers (the middle panel) of female BALB/c nude mice performed with tail vein injection or intrasplenic injection in sh-NC and sh-NASP groups. Black arrows represented metastatic lesions. The number of nodules was counted (right panel). **E** Photograph of lungs (left panel) and livers (the middle panel) of female BALB/c nude mice performed with tail vein injection or intrasplenic injection in LV-vector and LV-NASP groups. Black arrows represented metastatic lesions. The number of nodules was counted (right panel). **F** H&E staining of lung and liver in LV-vector and LV-NASP groups. T: tumor. **G** Schematic illustration showing the mechanism of NASP in promoting TNBC metastasis. *P<0.05, **P<0.01, ***P<0.001, ****P<0.0001.

To confirm the carcinogenic role of NASP in TNBC *in vivo*, the xenograft experiment was first performed. The BT549 cells which steadily expressed sh-NC, sh-NASP, LV-vector or LV-NASP were injected subcutaneously into BALB/c nude mice respectively. The knockdown efficiency of sh-NASP was detected by western blotting (Figure S4). The collected tumors in sh-NASP group significantly had lower volume and weight, while the collected tumors in LV-NASP group significantly had larger volume and weight than in LV-vector group, showing that NASP promoted TNBC growth *in vivo* (Figure 7A-7C). We then established TNBC lung metastatic model via tail vein injection and liver metastatic model via intrasplenic injection. The metastatic organs were harvested. As presented in Figure 7D and Figure 7E, the number of metastatic nodules in lung or liver were significantly reduced when NASP was knocked down in TNBC. On the contrast, the number of metastatic nodules were significantly higher when NASP was overexpressed in TNBC. Consistently, H&E staining showed that there was an increased number of micro-metastases in LV-NASP group compared to LV-vector group (Figure 7F).

Altogether, NASP promoted the growth and metastasis of TNBC via stabilizing YAP in USP15-dependent way (Figure 7G).

## Discussion

Previous studies reported that NASP was a chaperone of H3-H4 histone and played important role in histone homeostasis, modification and metabolism 6, 10. In malignant tumor, NASP takes part in the progression of tumors. Suppressing NASP caused decreased histone H3K9me1 on the promoter of anti-tumor genes, resulting in inhibiting hepatocellular carcinoma 10. NASP was the downstream target gene of microRNA-29c in gastric cancer 44. In prostate cancer, renal cancer and melanoma, NASP took part in regulating cell cycle 8, 9, 45. Huang et al reported that sNASP promoted 5-FU resistance via TRAF6/NF-κB pathway in breast cancer 11. However, the role of NASP in different breast cancer subtypes was not clear, and how tNASP affected breast cancer was ignored. In this study, we compared the expression of NASP among breast cancer subtypes and discovered that NASP was only highly expressed in TNBC. As for the potential reason for the high expression of NASP, we found that the RRM2 motif of SRSF1 interacted with the mRNA of NASP and elevated its stability. It has been reported that SRSF1 regulate the stability of mRNA by directly binding to the AU-rich element in the 3'-untranslated regions of mRNA 46. Further investigations are still required to explore the site of NASP mRNA that interact with SRSF1. We next analyzed the biological functions and clinical significance of NASP in TNBC and found that NASP promoted TNBC progression and metastasis, and the prognosis of TNBC patients with high expression of NASP was poorer. These findings provided a new insight into the role of NASP in TNBC, differing from the role of NASP in drug-resistance.

YAP is an important oncogene which is highly expressed in multiple tumors and co-activate the transcription of oncogenes with a range of transcription factors 47. Previous studies have reported that YAP not only induced the tumorigenesis and proliferation of tumors, but also facilitated tumor metastasis in various ways, such as promoting the migration, invasion, anchor-independent growth, and epithelial-mesenchymal transition (EMT) 29, 48-50. The activation of YAP barely participated in all major steps of tumor metastasis. Thus, YAP might be a potential therapeutic target in preventing tumor metastasis. In this study, we discovered that NASP stimulated the progression and metastasis of TNBC by promoting the stability of YAP, and the effect of NASP on stabilizing YAP was related to the ubiquitination of YAP. These findings showing that NASP might be a novel therapeutic target of TNBC, considering its oncogenic role and YAP-facilitating role in TNBC. In addition, these results also indicated that a certain kind of ubiquitination-related enzyme might take part in NASP-regulated stability of YAP. Thus, we searched for the potential ubiquitination-related enzymes.

USP15 acted as a DUB in several cancers, such as glioblastoma 51, breast cancer 39 and ovarian cancer 52. The substrates of USP15 includes p53 53, BARD1 54, BMI1 55, etc., and different substrates lead to variety roles of USP15 in multiple biological processes, such as transcription activation, cell cycle, gene stabilization, immune response and tumor progression 41. We noticed that USP15 was a potential interactor of both NASP and YAP, thus we hypothesized that NASP regulated the ubiquitination of YAP in a USP15-dependent way. Here we discovered that YAP was a substrate of USP15 in TNBC. The C269 is necessary for USP15 to catalyze the deubiquitylation of the substrates, and the mutation of this site will lead to inactivation of USP15 56. In this study, the deubiquitylation of YAP decreased when the C269 of USP15 was mutated, which was consistent with previous reports. Previous studies reported that K48- and K63-linked polyubiquitination existed in ubiquitination of YAP. K48-linked polyubiquitination is mainly related to proteasomal degradation, while K63-linked polyubiquitination is related to endocytosis, signaling activation and protein complex formation 13. Consistently, we discovered that USP15 prevented YAP from degradation by deubiquitylating the K48-linked rather than K63-linked polyubiquitination of YAP. Moreover, we validated that the effect of NASP on stabilizing YAP in TNBC depended on USP15, uncovering a novel mechanism in TNBC progression and metastasis.

In summary, this study suggested that NASP promotes TNBC progression and metastasis by stabilizing YAP in a USP15-dependent way (Figure 7G). It brought new insights to the mechanism of TNBC metastasis and provided potential therapeutic targets for preventing TNBC progression and metastasis.

## Supplementary Material

Supplementary figures and tables.

## Figures and Tables

**Table 1:** The relationships between NASP expression and clinical characteristics in TNBC patients.

Clinical characteristics	Total	NASP expression		*P* value
		High (N=40)	Low (N=40)	
Age at diagnosis				
<60	38	17	21	0.370
≥60	42	23	19	
Stage				
I and II	73	34	39	0.108
III and IV	7	6	1	
Lymph node status				
Negative	63	31	32	0.785
Positive	17	9	8	
Distant metastasis				
No	66	28	38	0.003^**^
Yes	14	12	2	

**P<0.01

## Abbreviations

CHX: cycloheximide
co-IP: coimmunoprecipitation
CTGF: connective tissue growth factor
DMFS: distant metastasis-free survival
DUB: deubiquitinating enzyme
EMT: epithelial-mesenchymal transition
H&E: hematoxylin and eosin
HER-2: human epidermal growth factor receptor-2
HR: hormone receptor
IF: immunofluorescence
IHC: immunohistochemistry
NASP: nuclear autoantigenic sperm protein
PCR: polymerase chain reaction
PFS: progression-free survival
RIP: RNA binding protein immunoprecipitation
RT: reverse transcription
RT-qPCR: real time quantitative PCR
SD: standard deviation
sNASP: somatic nuclear autoantigenic sperm protein
SRSF1: serine and arginine rich splicing factor 1
tNASP: testicular nuclear autoantigenic sperm protein
TNBC: triple-negative breast cancer
USP: ubiquitin specific peptidase
WT: wild-type
YAP: yes-associated protein

## References

[1] Sung H, Ferlay J, Siegel RL, Laversanne M, Soerjomataram I, Jemal A (2021). Global Cancer Statistics 2020: GLOBOCAN Estimates of Incidence and Mortality Worldwide for 36 Cancers in 185 Countries. CA: a cancer journal for clinicians.

[2] Nolan E, Lindeman GJ, Visvader JE (2023). Deciphering breast cancer: from biology to the clinic. Cell.

[3] Li Y, Li M, Su K, Zong S, Zhang H, Xiong L (2023). Pre-metastatic niche: from revealing the molecular and cellular mechanisms to the clinical applications in breast cancer metastasis. Theranostics.

[4] Li Y, Zhang H, Merkher Y, Chen L, Liu N, Leonov S (2022). Recent advances in therapeutic strategies for triple-negative breast cancer. Journal of hematology & oncology.

[5] Welch JE, O'Rand MG (1990). Characterization of a sperm-specific nuclear autoantigenic protein. II. Expression and localization in the testis. Biology of reproduction.

[6] Bao H, Carraro M, Flury V, Liu Y, Luo M, Chen L (2022). NASP maintains histone H3-H4 homeostasis through two distinct H3 binding modes. Nucleic acids research.

[7] Kato D, Osakabe A, Tachiwana H, Tanaka H, Kurumizaka H (2015). Human tNASP promotes in vitro nucleosome assembly with histone H3.3. Biochemistry.

[8] Alekseev OM, Richardson RT, Tsuruta JK, O'Rand MG (2011). Depletion of the histone chaperone tNASP inhibits proliferation and induces apoptosis in prostate cancer PC-3 cells. Reproductive biology and endocrinology: RB&E.

[9] Fang J, Wang H, Xi W, Cheng G, Wang S, Su S (2015). Downregulation of tNASP inhibits proliferation through regulating cell cycle-related proteins and inactive ERK/MAPK signal pathway in renal cell carcinoma cells. Tumour biology: the journal of the International Society for Oncodevelopmental Biology and Medicine.

[10] Kang X, Feng Y, Gan Z, Zeng S, Guo X, Chen X (2018). NASP antagonize chromatin accessibility through maintaining histone H3K9me1 in hepatocellular carcinoma. Biochimica et biophysica acta Molecular basis of disease.

[11] Huang Y, Yang S, Yu W, Gui L (2022). Somatic nuclear auto-antigenic sperm protein sensitizes human breast cancer cells to 5-Fluorouracil. Cancer chemotherapy and pharmacology.

[12] Mansour MA (2018). Ubiquitination: Friend and foe in cancer. The international journal of biochemistry & cell biology.

[13] Cockram PE, Kist M, Prakash S, Chen SH, Wertz IE, Vucic D (2021). Ubiquitination in the regulation of inflammatory cell death and cancer. Cell death and differentiation.

[14] Harrigan JA, Jacq X, Martin NM, Jackson SP (2018). Deubiquitylating enzymes and drug discovery: emerging opportunities. Nature reviews Drug discovery.

[15] Chen S, Liu Y, Zhou H (2021). Advances in the Development Ubiquitin-Specific Peptidase (USP) Inhibitors. International journal of molecular sciences.

[16] Young MJ, Hsu KC, Lin TE, Chang WC, Hung JJ (2019). The role of ubiquitin-specific peptidases in cancer progression. Journal of biomedical science.

[17] Kim D, Hong A, Park HI, Shin WH, Yoo L, Jeon SJ (2017). Deubiquitinating enzyme USP22 positively regulates c-Myc stability and tumorigenic activity in mammalian and breast cancer cells. Journal of cellular physiology.

[18] Sun XX, He X, Yin L, Komada M, Sears RC, Dai MS (2015). The nucleolar ubiquitin-specific protease USP36 deubiquitinates and stabilizes c-Myc. Proceedings of the National Academy of Sciences of the United States of America.

[19] Pan J, Deng Q, Jiang C, Wang X, Niu T, Li H (2015). USP37 directly deubiquitinates and stabilizes c-Myc in lung cancer. Oncogene.

[20] Zhang L, Zhou F, Drabsch Y, Gao R, Snaar-Jagalska BE, Mickanin C (2012). USP4 is regulated by AKT phosphorylation and directly deubiquitylates TGF-β type I receptor. Nature cell biology.

[21] Shi J, Zhang Q, Yin X, Ye J, Gao S, Chen C (2023). Stabilization of IGF2BP1 by USP10 promotes breast cancer metastasis via CPT1A in an m6A-dependent manner. International journal of biological sciences.

[22] Zheng W, Wang X, Yu Y, Ji C, Fang L (2023). CircRNF10-DHX15 interaction suppressed breast cancer progression by antagonizing DHX15-NF-κB p65 positive feedback loop. Cellular & molecular biology letters.

[23] Paz S, Ritchie A, Mauer C, Caputi M (2021). The RNA binding protein SRSF1 is a master switch of gene expression and regulation in the immune system. Cytokine & growth factor reviews.

[24] Sheng J, Zhao Q, Zhao J, Zhang W, Sun Y, Qin P (2018). SRSF1 modulates PTPMT1 alternative splicing to regulate lung cancer cell radioresistance. EBioMedicine.

[25] Martínez-Terroba E, Ezponda T, Bértolo C, Sainz C, Remírez A, Agorreta J (2018). The oncogenic RNA-binding protein SRSF1 regulates LIG1 in non-small cell lung cancer. Laboratory investigation; a journal of technical methods and pathology.

[26] Michlewski G, Sanford JR, Cáceres JF (2008). The splicing factor SF2/ASF regulates translation initiation by enhancing phosphorylation of 4E-BP1. Molecular cell.

[27] Cléry A, Krepl M, Nguyen CKX, Moursy A, Jorjani H, Katsantoni M (2021). Structure of SRSF1 RRM1 bound to RNA reveals an unexpected bimodal mode of interaction and explains its involvement in SMN1 exon7 splicing. Nature communications.

[28] Nguyen CDK, Yi C (2019). YAP/TAZ Signaling and Resistance to Cancer Therapy. Trends in cancer.

[29] Koo JH, Guan KL (2018). Interplay between YAP/TAZ and Metabolism. Cell metabolism.

[30] Liu Q, Li J, Zhang W, Xiao C, Zhang S, Nian C (2021). Glycogen accumulation and phase separation drives liver tumor initiation. Cell.

[31] Hasegawa K, Fujii S, Matsumoto S, Tajiri Y, Kikuchi A, Kiyoshima T (2021). YAP signaling induces PIEZO1 to promote oral squamous cell carcinoma cell proliferation. The Journal of pathology.

[32] Lee CK, Jeong SH, Jang C, Bae H, Kim YH, Park I (2019). Tumor metastasis to lymph nodes requires YAP-dependent metabolic adaptation. Science (New York, NY).

[33] Zhao M, Zhang Y, Jiang Y, Wang K, Wang X, Zhou D (2021). YAP promotes autophagy and progression of gliomas via upregulating HMGB1. Journal of experimental & clinical cancer research: CR.

[34] Wang X, Ji C, Hu J, Deng X, Zheng W, Yu Y (2021). Hsa_circ_0005273 facilitates breast cancer tumorigenesis by regulating YAP1-hippo signaling pathway. Journal of experimental & clinical cancer research: CR.

[35] Ye D, Wang Y, Deng X, Zhou X, Liu D, Zhou B (2023). DNMT3a-dermatopontin axis suppresses breast cancer malignancy via inactivating YAP. Cell death & disease.

[36] Kisselev AF (2021). Site-Specific Proteasome Inhibitors. Biomolecules.

[37] Schneider-Poetsch T, Ju J, Eyler DE, Dang Y, Bhat S, Merrick WC (2010). Inhibition of eukaryotic translation elongation by cycloheximide and lactimidomycin. Nature chemical biology.

[38] Shi C, Yang X, Hou Y, Jin X, Guo L, Zhou Y (2022). USP15 promotes cGAS activation through deubiquitylation and liquid condensation. Nucleic acids research.

[39] Sun X, Tang H, Chen Y, Chen Z, Hu Z, Cui Z (2023). Loss of the receptors ER, PR and HER2 promotes USP15-dependent stabilization of PARP1 in triple-negative breast cancer. Nature cancer.

[40] Chen LL, Smith MD, Lv L, Nakagawa T, Li Z, Sun SC (2020). USP15 suppresses tumor immunity via deubiquitylation and inactivation of TET2. Science advances.

[41] Li YC, Cai SW, Shu YB, Chen MW, Shi Z (2022). USP15 in Cancer and Other Diseases: From Diverse Functionsto Therapeutic Targets. Biomedicines.

[42] Liu M, Yan M, Lv H, Wang B, Lv X, Zhang H (2020). Macrophage K63-Linked Ubiquitination of YAP Promotes Its Nuclear Localization and Exacerbates Atherosclerosis. Cell reports.

[43] Zhou X, Li Y, Wang W, Wang S, Hou J, Zhang A (2020). Regulation of Hippo/YAP signaling and Esophageal Squamous Carcinoma progression by an E3 ubiquitin ligase PARK2. Theranostics.

[44] Yu B, Chen X, Li J, Gu Q, Zhu Z, Li C (2017). microRNA-29c inhibits cell proliferation by targeting NASP in human gastric cancer. BMC cancer.

[45] Li JX, Wei CY, Cao SG, Xia MW (2019). Elevated nuclear auto-antigenic sperm protein promotes melanoma progression by inducing cell proliferation. OncoTargets and therapy.

[46] Delestienne N, Wauquier C, Soin R, Dierick JF, Gueydan C, Kruys V (2010). The splicing factor ASF/SF2 is associated with TIA-1-related/TIA-1-containing ribonucleoproteic complexes and contributes to post-transcriptional repression of gene expression. The FEBS journal.

[47] Totaro A, Panciera T, Piccolo S (2018). YAP/TAZ upstream signals and downstream responses. Nature cell biology.

[48] Liu M, Zhao S, Lin Q, Wang XP (2015). YAP regulates the expression of Hoxa1 and Hoxc13 in mouse and human oral and skin epithelial tissues. Molecular and cellular biology.

[49] Warren JSA, Xiao Y, Lamar JM (2018). YAP/TAZ Activation as a Target for Treating Metastatic Cancer. Cancers.

[50] Zhao W, Wang M, Cai M, Zhang C, Qiu Y, Wang X (2021). Transcriptional co-activators YAP/TAZ: Potential therapeutic targets for metastatic breast cancer. Biomedicine & pharmacotherapy = Biomedecine & pharmacotherapie.

[51] Eichhorn PJ, Rodón L, Gonzàlez-Juncà A, Dirac A, Gili M, Martínez-Sáez E (2012). USP15 stabilizes TGF-β receptor I and promotes oncogenesis through the activation of TGF-β signaling in glioblastoma. Nature medicine.

[52] Padmanabhan A, Candelaria N, Wong KK, Nikolai BC, Lonard DM, O'Malley BW (2018). USP15-dependent lysosomal pathway controls p53-R175H turnover in ovarian cancer cells. Nature communications.

[53] Liu WT, Huang KY, Lu MC, Huang HL, Chen CY, Cheng YL (2017). TGF-β upregulates the translation of USP15 via the PI3K/AKT pathway to promote p53 stability. Oncogene.

[54] Peng Y, Liao Q, Tan W, Peng C, Hu Z, Chen Y (2019). The deubiquitylating enzyme USP15 regulates homologous recombination repair and cancer cell response to PARP inhibitors. Nature communications.

[55] Zhang L, Qiang J, Yang X, Wang D, Rehman AU, He X (2020). IL1R2 Blockade Suppresses Breast Tumorigenesis and Progression by Impairing USP15-Dependent BMI1 Stability. Advanced science (Weinheim, Baden-Wurttemberg, Germany).

[56] Ward SJ, Gratton HE, Indrayudha P, Michavila C, Mukhopadhyay R, Maurer SK (2018). The structure of the deubiquitinase USP15 reveals a misaligned catalytic triad and an open ubiquitin-binding channel. The Journal of biological chemistry.

