# Supplementary material for: NASP Promotes Triple-negative Breast Cancer Progression and Metastasis by Stabilizing YAP in a USP15-Dependent Way
Source: Int J Biol Sci. 2025 Jun 20;21(9):4172–86. doi: 10.7150/ijbs.99438 (PMC12223775; doi:10.7150/ijbs.99438)
Supplement: Supplementary file 1 — Supplementary figures and tables. [file ijbsv21p4172s1.pdf]

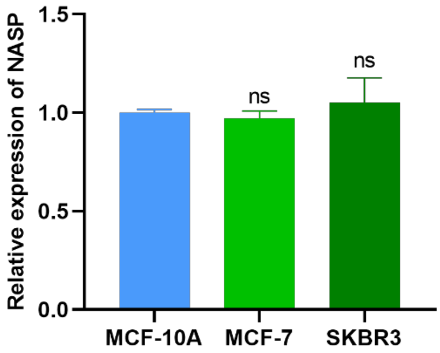

Figure S1. NASP expression in luminal (MCF-7) and Her2+ (SKBR3) breast cancer cells detected by RT-qPCR.

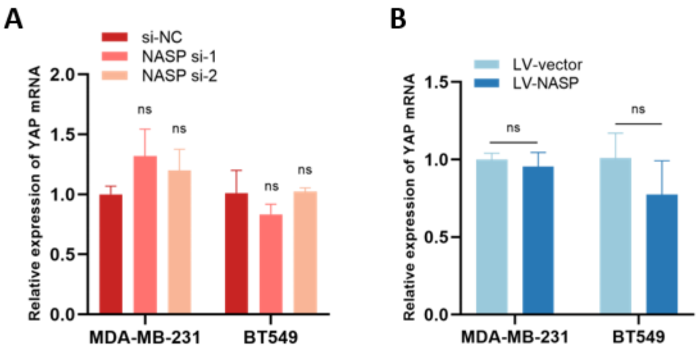

Figure S2. NASP did not affect the expression of YAP mRNA in TNBC. A The effect of interfering NASP on the expression of YAP mRNA. **B** The effect of overexpressing NASP on the expression of YAP mRNA. ns: no significance.

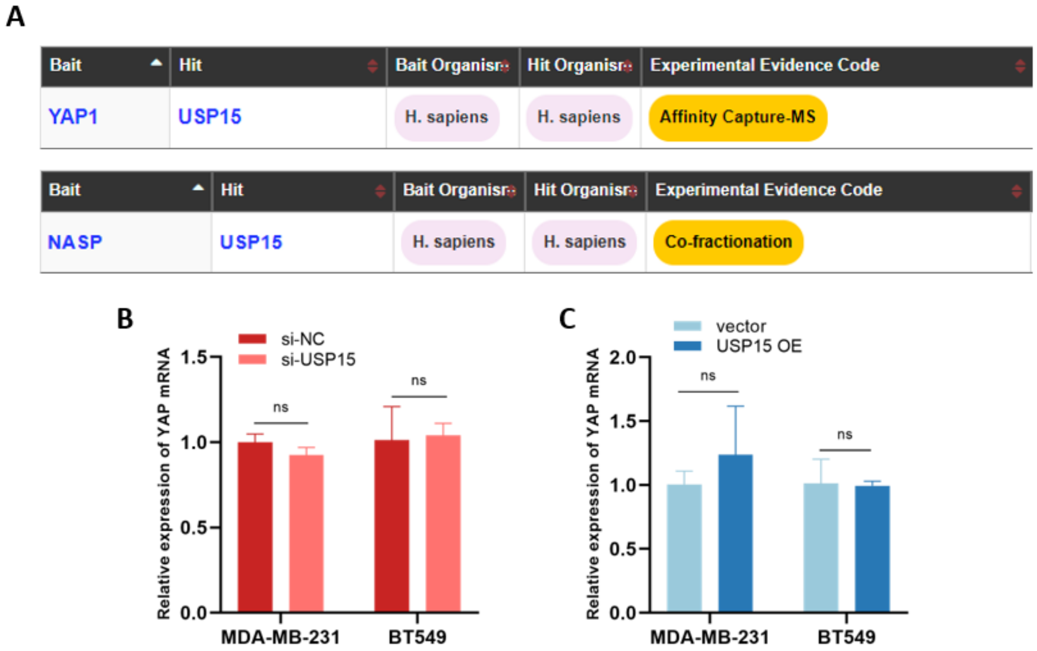

Figure S3. USP15 did not affect the expression of YAP mRNA in TNBC. A The potential interactions between YAP and USP15, NASP and USP15 predicted in BioGRID database. **B** The effect of interfering USP15 on the expression of YAP mRNA. **C** The effect of overexpressing USP15 on the expression of YAP mRNA. ns: no significance.

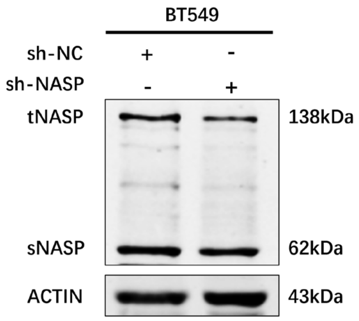

Figure S4. The knockdown efficiency of sh-NASP in BT549 cells.

Table S1. The sequences of siRNAs.

siRNAs	Sequences (5'-3')
si-NC sense	UUCUCCGAACGUGUCACGUTT
si-NC anti-sense	ACGUGACACGUUCGGAGAATT
NASP si-1 sense	GGAAAUCACUUCUGGAGUUTT
NASP si-1 anti-sense	AACUCCAGAAGUGAUUUCCTT
NASP si-2 sense	GAUCGUCUGCUGAAUACAATT
NASP si-2 anti-sense	UUGUAUUCAGCAGACGAUCTT
si-SRSF1 sense	GCUGAUGUUUACCGAGAUGTT
si-SRSF1 anti-sense	CAUCUCGGUAAACAUCAGCTT
si-USP15 sense	AGTCGCTGGTTCAAACAGTTT
si-USP15 anti-sense	ACUGUUUGAACCAGCGACUTT
si-YAP sense	GCGUAGCCAGUUACCAACATT
si-YAP anti-sense	UGUUGGUAACUGGCUACGCTT

Table S2. The Sequences of primers.

Primers	Sequences (5'-3')
NASP-Forward	GAAAACTATGTGCAAGCTGTGG
NASP-Reverse	ACTGAGAGTTGTACCCATAAGCC
YAP-Forward	TAGCCCTGCGTAGCCAGTTA
YAP-Reverse	TCATGCTTAGTCCACTGTCTGT
USP15-Forward	CGACGCTGCTCAAAACCTC
USP15-Reverse	TCCCATCTGGTATTTGTCCCAA
SRSF1-Forward	ATTCCTGCCCCAACCAAACC
SRSF1-Reverse	TGCTCCAGCGTTTATCTCCA
ACTB-Forward	GATCATTGCTCCTCCTGAGC
ACTB-Reverse	ACTCCTGCTTGCTGATCCAC
